# Trends in the diagnostic prevalence of cannabis-related disorders and co-occurring psychiatric disorders in adolescents: analysis of German health insurance data from 2013 to 2022

**DOI:** 10.1093/eurpub/ckaf228

**Published:** 2026-01-09

**Authors:** Alexander Zarour, Christian Bachmann, Lisa Dandolo, Jakob Holstiege, Falk Hoffmann, Constanze Scholman, Yulia Golub

**Affiliations:** Department of Child and Adolescent Psychiatry, Psychosomatics and Psychotherapy, School of Medicine and Health Sciences, Carl von Ossietzky Universität Oldenburg, Oldenburg, Germany; Department of Child and Adolescent Psychiatry, University Hospital Ulm, Ulm, Germany; Department of Child and Adolescent Psychiatry, Psychosomatics and Psychotherapy, School of Medicine and Health Sciences, Carl von Ossietzky Universität Oldenburg, Oldenburg, Germany; Department of Epidemiology and Healthcare Atlas, Central Research Institute of Ambulatory Health Care in Germany, Berlin, Germany; Division of Outpatient Care and Pharmacoepidemiology, Department of Health Services Research, Carl von Ossietzky Universität Oldenburg, Oldenburg, Germany; Department of Child and Adolescent Psychiatry, Psychosomatics and Psychotherapy, School of Medicine and Health Sciences, Carl von Ossietzky Universität Oldenburg, Oldenburg, Germany; Department of Child and Adolescent Psychiatry, Psychosomatics and Psychotherapy, School of Medicine and Health Sciences, Carl von Ossietzky Universität Oldenburg, Oldenburg, Germany

## Abstract

Cannabis use can have detrimental effects on adolescents’ mental health, often co-occurring with child and adolescent psychiatric disorders (CAPD). This study assessed diagnostic prevalence trends in cannabis-related disorders and co-occurring diagnosed CAPD in adolescents receiving outpatient treatment in Germany. Outpatient claims data from the national public health insurance system, covering almost 4 million children and adolescents, were assessed for diagnostic prevalence of cannabis-related disorders (ICD-10 diagnoses F12.X) in insurees aged 12 to 17 years for the years 2013–22, stratified by age group and sex. In addition, the diagnostic prevalence of co-occurring CAPD during the year 2022 was evaluated. From 2013 to 2022, the diagnostic prevalence of cannabis-related disorders among German adolescents utilizing outpatient services increased from 0.08% to 0.10% (+22.4%), with a decline during the COVID-19 pandemic and a higher diagnostic prevalence in older adolescents. Up to 14 years of age, the diagnostic prevalence of cannabis-related disorders was distributed evenly among males and females, while from age 15 onwards, the diagnostic prevalence was higher in males. Overall, 78.3% of adolescents diagnosed with cannabis-related disorders had at least one co-occurring CAPD diagnosis in 2022. Most common co-occurring conditions were depressive disorders, conduct disorders, adjustment disorders, attention-deficit/hyperactivity disorders, and anxiety disorders. Co-occurring depression was particularly often diagnosed, underscoring the urgent need for integrated treatment approaches addressing both disorders simultaneously in this age group.

## Introduction

Cannabis use has a high prevalence in adolescents, with 12.8% of all 12- to 17-year-olds reporting its use in 2019 in the USA [1]. In Germany, 9.3% of adolescents aged 12–17 years old indicated having used cannabis at least once in their lifetime, with 7.6% reporting cannabis use in the last year [2].

According to a large representative sample from the general population in Germany, 2.6% of adolescents aged 12–18 years meet the criteria for at least one cannabis use disorder (CUD), and 0.8% meet the criteria of cannabis dependence [3]. Of all adult cannabis users, 9% develop cannabis dependence [4]. When adolescents use cannabis, they are 2–4 times more likely to develop cannabis dependence than users starting above 18 years old [5].

Besides CUD, cannabis use in adolescence is associated with a range of co-occurring child and adolescent psychiatric disorders (CAPD), such as depressive disorders with higher rates of suicidality, psychosis, anxiety, attention-deficit/hyperactivity disorder (ADHD), and personality disorders, as well as post-traumatic stress disorder [6–12]. A nationally representative comorbidity survey of adolescents aged 13–18 years in the USA (*N* = 10 123) presented alarming results showing increased risk of depression and more severe depressive symptoms during adolescence due to cannabis use [13]. A meta-analysis demonstrated that cannabis use increases the risk of a suicide attempt, suicidal ideation, and suicide planning in young individuals of 11–21 years of age [14].

The relationship between cannabis use and psychiatric disorders is suggested to be bidirectional: while cannabis use can increase the risk of developing psychiatric conditions, adolescents with existing CAPD are also more likely to use cannabis [15]. The self-medication hypothesis suggests that individuals may use cannabis to cope with various psychiatric symptoms, relying on its perceived ability to alleviate emotional distress or regulate mood [15].

In comparison to the general population, rates of cannabis use and CUD are significantly elevated among those with psychiatric conditions [15]. Lev-Ran *et al*. [16] reported that individuals with a psychiatric disorder were almost 10 times as likely to use cannabis weekly or suffer from a CUD, compared to individuals without a mental health disorder [16].

In Germany, the prevalence of cannabis use in adults aged 18–59 raised from 4.4% in 1995 to 10% in 2021. The prevalence of heavy users remained relatively stable from 1995 (11.4%) to 2018 (9.5%), but increased to 15.7% during the COVID-19 pandemic in 2021 [17].

Since July 2024, the Cannabis Act has been in Germany, which aims to control the supply of cannabis for recreational use for adults [18]. Cannabis use and possession remain illegal for minors in Germany; however, the legalization of the drug for adults may reduce perceived health risks, potentially leading to an increased use among adolescents and a rise in the prevalence of CUD [19]. For instance, in Canada, where cannabis has been legal for adults for over 5 years, studies have shown a significant increase in cannabis use across the population, including minors [20, 21]. Thus, cannabis-related disorders are becoming an increasing concern among adolescents, yet there is limited knowledge regarding the number of young individuals diagnosed with cannabis-related disorders in Germany.

This study aims to address this gap by analyzing trends in outpatient diagnoses of cannabis-related disorders among adolescents aged 12–17 years old from 2013 to 2022. It also identifies the most common diagnosis of co-occurring CAPD associated with diagnosed cannabis-related disorders in this population in 2022.

## Methods

### Data source

The current dataset reflects outpatient healthcare service utilization among patients covered by the German statutory health insurance in accordance with § 295 of the German Social Code (SGB, Sozialgesetzbuch). In Germany, 87% of the population are covered by statutory health insurance—equivalent to around 72 million people [22]. The German healthcare system follows the principle that all necessary medical, psychotherapeutic, and psychiatric services are free and accessible for children and adolescents. There are three main pillars covered by the statutory health insurance reflecting the stepwise services withing the health care routs for patients with substance use disorders (SUD)—beginning with (i) initial access services (outpatient pediatricians, general practitioners, child and adolescent psychotherapists and child and adolescent psychiatrists), followed by (ii) specialized outpatient services (child and adolescent psychiatric and psychotherapeutic counseling and treatment provided for therapeutic purposes and preparation for inpatient treatment or part of post-inpatient aftercare), and, when indicated, (iii) inpatient services (qualified withdrawal in general child and adolescent psychiatric wards or qualified withdrawal, rehabilitation programs and treatment of comorbid psychiatric disorders in specialized child and adolescent addiction psychiatric wards). This dataset comprises data from all outpatient health services (OHS).

### Diagnostic prevalence from outpatient claims data

Diagnostic prevalence of cannabis-related disorders was retrieved from an outpatient claims database, comprising all adolescents insured by statutory health insurance and utilizing the OHS at least once in each respective year. Importantly, the measure of diagnostic prevalence describes the number of diagnoses given within the health care system, mainly for reasons of documentation and billings, and therefore cannot be directly related to the prevalence of a mental disorder in this population [23]. Thus, existing cannabis-related disorders may not always be recorded when individuals utilize OHS for other medical reasons and do not actively report issues resulting from cannabis use.

### Classification of cannabis-related disorders

In the OHS, diagnoses are recorded based on the International Classification of Diseases, Tenth Revision (ICD-10). This study specifically focused on adolescents diagnosed with the ICD-10 code F12, which pertains to mental and behavioral disorders resulting from cannabis use. F12 diagnoses can be further subdivided into the categories F12.0 (acute intoxication), F12.1 (harmful use), F12.2 (dependence syndrome), F12.3 (withdrawal state), F12.4 (withdrawal state with delirium), F12.5 (psychotic disorder), F12.6 (amnesic syndrome), F12.7 (residual and late-onset psychotic disorder), F12.8 (other disorders), and F12.9 (unspecified disorder).

### Time trends and groups

Diagnostic prevalence was available for the years 2013–22 for adolescents aged 12–17 years old. These adolescents were stratified into two age groups: 12–14 years old and 15–17 years old. Sex was analyzed among male and female-identifying youth.

### Co-occurring CAPD diagnoses

For analyzing co-occurring CAPD diagnoses, cross-sectional data were available from the year 2022. The following co-occurring CAPD disorders were included as in previous studies [24]: psychotic disorders (F20–F22, F25), bipolar disorders (F30, F31), depressive disorders (F32, F33, F41.2), other mood disorders (F34, F38, F39), anxiety disorders/emotional disorders (F40, F41, F93), obsessive–compulsive disorder (F42), reaction to severe stress, and adjustment disorders (F43), eating disorders (F50), sleeping disorders (F51, G47), personality disorders (F60–F69), mental retardation (F70–F79, F84.4), autism spectrum disorder (F84.0/1/5/8/9), ADHD (F90, F98.8), conduct disorders (F90.1, F91, F92), attachment disorders (F94.1, F94.2), and tic disorders (F95) [24]. For each of these CAPD disorders, we first calculated the diagnostic prevalence within the group of adolescents with cannabis-related disorders (*n* = 3701 in the year 2022). We additionally calculated the diagnostic prevalence for the same CAPD disorders in a 1:10 age- and sex-matched control group of adolescents without a F12 diagnosis (*n* = 37 010).

### Co-occurring diagnoses of other substance-related disorders

For the group of adolescents with cannabis-related disorders in the year 2022 (*n* = 3701), we additionally report the diagnostic prevalence of other co-occurring substance-related disorders: alcohol (F10), opioids (F11), sedatives or hypnotics (F13), cocaine (F14), other stimulants including caffeine (F15), hallucinogens (F16), tobacco (F17), volatile solvents (F18), and multiple drug use and use of other psychoactive substances (F19).

## Results

### Trends in diagnostic prevalence of cannabis-related disorders among adolescents utilizing OHS in Germany (2013–22)

Each calendar year, data of more than 3.6 million children and adolescents were available (e.g. 2013: 3 934 313 children and adolescents, 2022: 3 819 821 children and adolescents). Overall, in 2022, 3701 adolescents were diagnosed with cannabis-related disorders, corresponding to a diagnostic prevalence of 0.1%. The data across time showed that the diagnostic prevalence of cannabis-related disorders among adolescents had risen from 0.08% in 2013 to 0.13% in 2019. Over the following 2 years, during the COVID-19 pandemic, the diagnostic prevalence decreased from 0.12% in 2020 to 0.10% in 2022. Nevertheless, there was an increase in diagnostic prevalence of 22.4% from the year 2013 to 2022.

**Figure 1.**
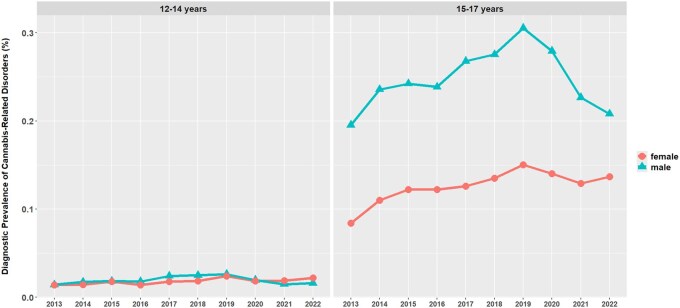
Diagnostic prevalence of cannabis-related disorders (%) among adolescents utilizing the outpatient health service from the years 2013 to 2022. Left panel: age 12–14 years old, right panel: age 15–17 years old, triangles = male, circles = female.

As can be seen in Fig. 1, more than 90% of patients diagnosed with cannabis-related disorders were 15–17 years old (e.g. 2013: 91.5%, 2022: 90.4%). In this age group, the diagnostic prevalence of cannabis-related disorders was higher in male adolescents than in female adolescents (e.g. 2013: diagnostic prevalence in males 0.20%, in females 0.08%, corresponding to a distribution of 69.9% males to 30.1% females; 2022: diagnostic prevalence in males 0.21%, in females 0.14%, corresponding to a distribution of 60.3% males to 39.7% females; Fig. 1). In the age group of 12–14 years, the diagnostic prevalence of cannabis-related disorders was rather low in general and similar for male and female adolescents (2013: 0.01% diagnostic prevalence in males, 0.01% in females, corresponding to a distribution of 50.2% males to 49.8% females; 2022: 0.02% diagnostic prevalence in males, 0.02% in females).

Among all F12 diagnoses, the subcategory F12.1 indicating harmful use was by far the most often diagnosed (e.g. in the year 2022: 73.0% of all diagnoses), followed by the diagnosis of dependence syndrome F12.2 (19.7%) (see Supplementary Fig. S1).

### Co-occurring CAPD in the year 2022

**Figure 2.**
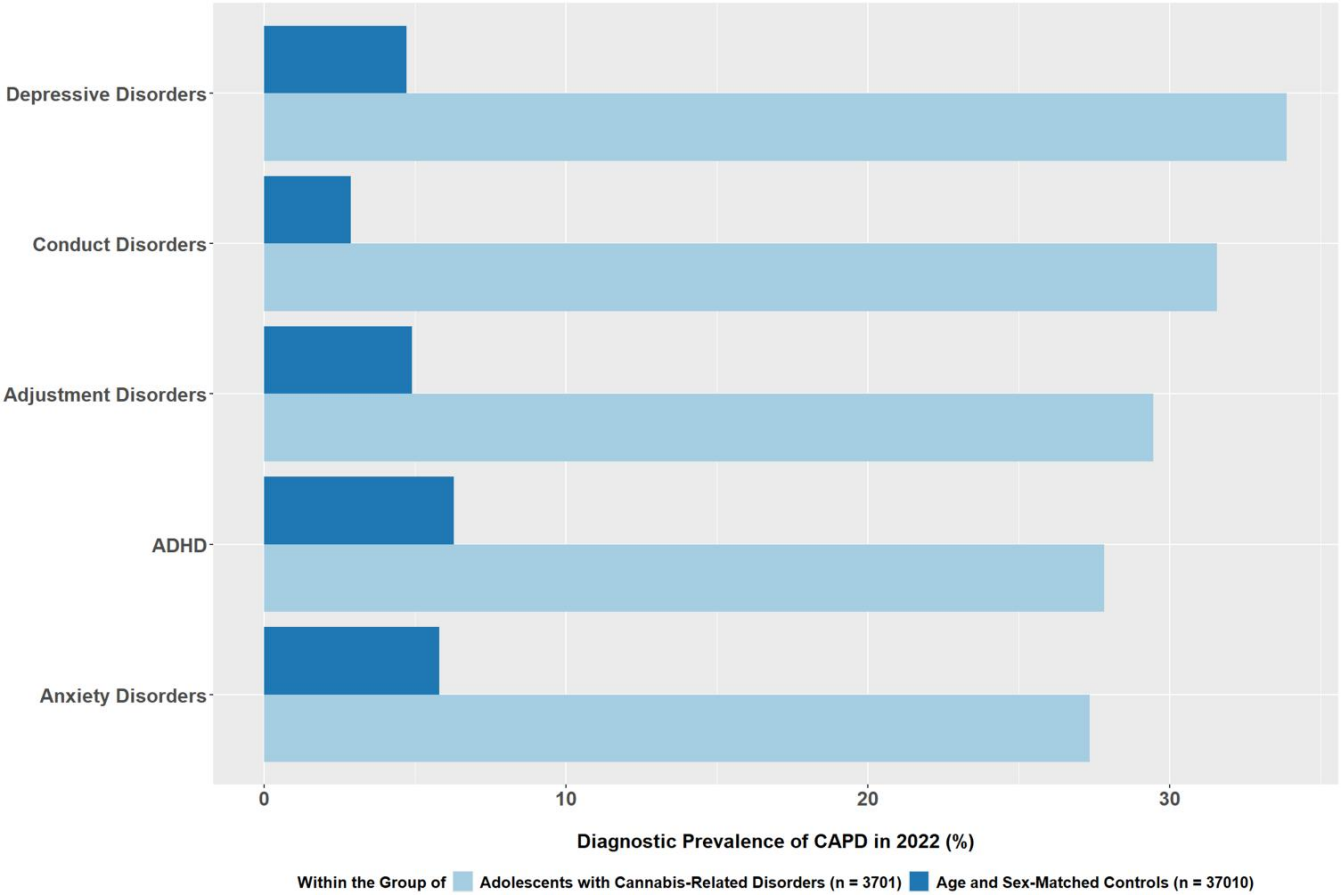
Diagnostic prevalence of the five most common co-occurring CAPD in 2022 within the group of adolescents with a diagnosis of cannabis-related disorders (*n *= 3701, light bars) and within a 1:10 age- and sex-matched control group of adolescents without a F12 diagnosis (*n* = 37 010, dark bars).

In the group of adolescents with cannabis-related disorders, at least one other CAPD disorder was diagnosed in 78.3% of the patients. Alarmingly, 32.2% of all patients had three or more co-occurring CAPD. In comparison, in a 1:10 age- and sex-matched control group of adolescents without a F12 diagnosis, only 19.1% were diagnosed with at least one CAPD, and only 3.3% had three or more co-occurring CAPD (see Supplementary Fig. S2). As can be seen in Table 1, for each of the included CAPD categories, the diagnostic prevalence was considerably higher in the group of adolescents with cannabis-related disorders than in the control group. Figure 2 visualizes the five most common CAPD, with depressive disorders diagnoses being the most prevalent within the group of adolescents with cannabis-related disorders affecting around one third of these patients (33.9%). Other common CAPD diagnoses in this group were conduct disorders (31.6%), adjustment disorders (29.5%), ADHD (27.8%) and anxiety disorder (27.3%) (Fig. 2). A direct comparison to the five most common CAPD in the control group showed that the diagnostic prevalence was not only lower in each of the categories, but also showed a slightly different pattern: here the diagnostic prevalence was highest for ADHD (6.3%), followed by anxiety disorders (5.8%), adjustment disorders (4.9%), depressive disorders (4.7%) and conduct disorders (2.9%) (Fig. 2).

**Table 1.** Total number of patients and diagnostic prevalence (%) of co-occurring CAPD in 2022 in adolescents with cannabis-related disorders (*n* = 3701) and a 1:10 age- and sex-matched control group without a F12 diagnosis (*n* = 37 010)

	Adolescents with cannabis-related disorders (*n* = 3701)	Age- and sex-matched controls (*n* = 37 010)
Total patients with any co-occurring CAPD	2898 (78.3)	7079 (19.1)
Patients with one co-occurring CAPD	875 (23.6)	3938 (10.6)
Patients with two co-occurring CAPD	833 (22.5)	1910 (5.2)
Patients with three or more co-occurring CAPD	1190 (32.2)	1231 (3.3)
Co-occurring CAPD (ICD-10)		
Depressive disorders (F32, F33, F41.2)	1254 (33.9)	1748 (4.7)
Conduct disorders (F90.1, F91, F92)	1168 (31.6)	1064 (2.9)
Reaction to severe stress, and adjustment disorders (F43)	1090 (29.5)	1810 (4.9)
Hyperkinetic disorders (F90, F98.8)	1030 (27.8)	2326 (6.3)
Anxiety disorders/emotional disorders (F40, F41, F93)	1012 (27.3)	2150 (5.8)
Personality disorders (F60–F69)	473 (12.8)	656 (1.8)
Sleeping disorders (F51, G47)	315 (8.5)	530 (1.4)
Eating disorders (F50)	135 (3.7)	356 (1.0)
Other mood disorders (F34, F38, F39)	102 (2.8)	109 (0.3)
Attachment disorders (F94.1, F94.2)	79 (2.1)	82 (0.2)
Autism spectrum disorder (F84.0/1/5/8/9)	69 (1.9)	425 (1.2)
Tic disorders (F95)	52 (1.4)	148 (0.4)
Psychotic disorders (F20–F22, F25)	48 (1.3)	30 (0.1)
Obsessive–compulsive disorder (F42)	42 (1.1)	187 (0.5)
Mental retardation (F70–F79, F84.4)	41 (1.1)	328 (0.9)

Furthermore, the adolescents with cannabis-related disorders were often also diagnosed with other substance-related disorders, especially with multiple drug use F19 (19.9%), tobacco F17 (16.0%), and alcohol F10 (15.5%) (see Supplementary Fig. S3).

## Discussion

First, we found a consistent rise in the number of male and female adolescents diagnosed with cannabis-related disorders within the OHS, peaking in 2019 (+52.3% compared to 2013), followed by a drop in 2020–22 during the COVID-19 pandemic. Overall, diagnoses of cannabis-related disorders in adolescents utilizing the OHS increased from 2013 to 2022 by 22.4%.

Second, among the adolescents diagnosed with mental and behavioral disorders due to cannabis use, 78.3% had co-occurring CAPD in 2022, with 32.2% diagnosed with three or more co-occurring CAPD. Depression (33.9% of patients) was the most common, followed by conduct disorders, adjustment disorders, ADHD, and anxiety disorders.

The diagnostic prevalence of cannabis-related disorders among adolescents utilizing the OHS (0.08% in 2013 and 0.10% in 2022) stands in a significant contrast with the prevalence of CUD reported in large population-representative studies in Germany. In a recent epidemiological study by Arnaud *et al*. [3], adolescents aged 12–18 years showed a prevalence of 2.6% for CUD in 2020, with 0.8% meeting the criteria for cannabis dependence [3]. When comparing our diagnostic prevalence data to these national prevalences, it seems like only about 3.5% of adolescents with CUD are diagnosed with cannabis-related disorders in the OHS.

### Trends during the COVID-19 pandemic and age and sex characteristics of diagnoses of cannabis-related disorders in adolescents

The number of patients diagnosed with cannabis-related disorders decreased during the COVID-19 pandemic in 2020 and 2021 compared to the years before (Fig. 1). We presume that the COVID-19 pandemic had strong effects on reducing the utilization of the outpatient healthcare services. Hoerold *et al*. [25] demonstrated that healthcare professionals experienced less consultations during COVID-19 in Germany [25].

Male patients diagnosed with cannabis-related disorders outnumbered females in the 15- to 17-year-old age group. These results are supported by other studies; for instance, Arnaud *et al*. [3] describe a similar pattern of male sex and older age being positively and statistically significantly associated with cannabis abuse and cannabis dependence. Male adolescents were 3.5 times more likely to meet criteria for cannabis dependence than females [3].

### Co-occurring child and adolescent psychiatric disorders

Our results show that depressive disorders are the most commonly observed co-occurring CAPD among adolescents diagnosed with cannabis-related disorders in the OHS. The diagnostic prevalence of depressive disorders within this group (33.9%) was about seven times higher than in a matched control group (4.7%). These data are in line with alarming results of the National Comorbidity Survey from the USA (*N* = 10 123; adolescents aged 13–18 years), which showed a higher risk of depression with more severe symptoms during adolescence due to cannabis use [26]. Additionally, the cannabinoid use could modify the serotonin system’s responsiveness, which could contribute to depression [27]. Alarmingly, both depression and cannabis use were demonstrated to be independently associated with higher odds of suicide attempts among adolescents [13]. It is known that either cannabis use could lead to a CAPD or a mental health disorder could lead to the use of cannabis [15]. In our dataset, in the year 2022, 0.9% of the patients diagnosed with depressive disorders (F32, F33, F41.2) were also diagnosed with cannabis-related disorders (F12), this is a nine times higher diagnostic prevalence of cannabis-related disorders than in our general sample (0.1% in 2022). Nevertheless, comparing it to the reverse figure—indicating that around one third of patients (33.9%) diagnosed with cannabis-related disorders were also diagnosed with depressive disorders—suggests that depressive symptoms are a much more prominent issue in patients with diagnosed cannabis-related disorders than issues resulting from cannabis use are in patients with diagnosed depressive disorders. This said, it might well be that many adolescents with depressive disorders show a harmful use of cannabis, without receiving an F12 diagnosis.

Conduct disorder was the second most commonly diagnosed co-occurring CAPD disorder within the group of adolescents diagnosed with cannabis-related disorders. Other studies showed patients with conduct disorder to have a higher risk of cannabis use compared to other psychiatric illnesses [28]. Arnaud *et al*. [3] revealed a strong association of SUD with impulsive personality traits, sensation seeking, and externalizing psychopathological symptoms in adolescents [3].

ADHD is also a common co-occurring child psychiatric disorder in our sample. A recent meta-analysis estimated the prevalence of ADHD among grown-up individuals with SUD at 21%, highlighting ADHD as a common comorbidity associated with an increased risk and earlier onset of severe SUD [29]. The study notes that the exact reason for the increased association between ADHD and SUD is unknown. It is suggested that substance abuse represents an attempt to self-medicate ADHD symptoms. Another study suggests that the increased prevalence of ADHD and SUD is the result of a developmental interaction between ADHD symptoms (e.g. impulsivity or behavior dysregulation) and the consequences of ADHD (e.g. poor academic performance), thereby increasing the risk of developing a SUD [30].

Overall, our results suggest that adolescents with cannabis-related disorders are at an increased risk for psychiatric conditions, with 32.2% receiving three or more diagnoses of CAPD, which necessitates treatment that includes both SUD treatment and treatment of co-occurring CAPD.

### Barriers to accessing outpatient health service due to CAPD

When comparing our data to national German statistics [3], it seems that only about 3.5% of adolescents with CUD are diagnosed with cannabis-related disorders in the OHS. Of course, our data reflect the diagnostic prevalence of cannabis-related disorders based on the ICD-10 in adolescents utilizing OHS, while the study by Arnaud *et al*. [3] measures the prevalence of CUD in a general population. Thus, we do not expect these numbers to align. Yet, it is important to discuss which factors might contribute to this low percentage of diagnoses of cannabis-related disorders in OHS.

It might be that not all adolescents with CUD feel like they need treatment, or they might discontinue the harmful use of cannabis through other types of help, like social services, helplines, or youth services. Nevertheless, the significant discrepancy between the prevalence of CUD in adolescents and the diagnostic prevalence of cannabis-related disorders in the OHS might also be a reflection of the individual and systemic barriers in accessing mental health care. It underscores the need for targeted interventions to bridge this gap, with individual, societal, and treatment service factors influencing access to care [31].

First, individual barriers might include lack of knowledge of symptoms and consequences of chronic cannabis use, lack of motivation, insufficient insight into the illness, and a general reluctance to seek help due to misconceptions about the nature of addiction [32]. Additionally, the lack of awareness among adolescents about available services contributes to underutilization. Second, societal barriers, such as the stigma associated with substance abuse or the normalization of cannabis use and cannabis-related disorders in certain communities, further discourage adolescents from seeking help. Third, the requirement for minors to be accompanied by a legal guardian when seeking healthcare services can further complicate access. Fourth, the awareness of healthcare facilitators like the general practitioner and pediatricians could be a barrier in diagnosing cannabis-related disorders in adolescents. The focus during consultations might be biased, concentrating solely on other psychiatric symptoms and not addressing cannabis use and cannabis-related disorders. Additionally, there may be a tendency to focus on “chemical” substances, which are often considered more “dangerous,” overlooking the growing concern of cannabis use.

The support for adolescents with cannabis-related disorders should include low-threshold counseling services, outpatient diagnostics and therapy, qualified inpatient detoxification and withdrawal treatment, as well as the treatment of co-occurring mental health disorders and rehabilitation treatment [33]. Currently, the availability of outpatient, as well as inpatient, and rehabilitation places for children and adolescents in Germany is insufficient, with many regions lacking appropriate services altogether [33]. This is especially alarming in light of the cannabis legalization act, which is expected to lead to an increase in cannabis use among adolescents and, consequently, in the prevalence of cannabis-related disorders such as CUD.

### Strengths and limitations

This study benefits from a large, representative dataset covering approximately 90% of the German population, providing a comprehensive overview of OHS utilization for cannabis-related disorders among adolescents over a 10-year period. Additionally, stratified analysis by age and sex allowed for detailed subgroup insights.

The retrospective design relies on pre-existing administrative data, which may be subject to coding errors or incomplete records and which did not allow to include additional assessment instruments. Co-occurring CAPD were only analyzed for 2022, limiting longitudinal insights into trends. Furthermore, the study excluded adolescents not covered by statutory health insurance, potentially underrepresenting specific demographic groups. Another limitation of this study is the absence of data on hospital visits, focusing only on outpatient care, which misses an important aspect of healthcare utilization. Additionally, it is important to stress that this study reports diagnostic prevalence, which should not be mistaken with the actual prevalence of the disorder in this population, as in many cases, patients seeking OHS for other kinds of medical symptoms are not asked about cannabis use.

## Conclusion

This study highlights a steady increase in diagnostic prevalence of cannabis-related disorders among adolescents utilizing the OHS in Germany from 2013 to 2019/2022, with a notable decline during the COVID-19 pandemic. The striking discrepancy between the prevalence of CUD in adolescents in Germany reported in studies over the same period of time and the diagnostic prevalence of cannabis-related disorders in adolescents utilizing the OHS might reflect substantial individual and systemic barriers to accessing mental health care. Depressive disorders are the most common co-occurring diagnosis among adolescents diagnosed with cannabis-related disorders, which aligns with US data indicating an increased risk of depression and suicidality among minors with CUD, underscoring the urgent need for integrated treatment approaches addressing both substance use and co-occurring psychiatric conditions.

## Supplementary Material

ckaf228_Supplementary_Data

## Data Availability

The data analyzed in this study are available upon request to the “Zi—Zentralinstitut kassenärztliche Versorgung” (https://www.zi.de/). Key points Outpatient data from the national statutory health insurance system, covering almost 4 million children and adolescents, were assessed for diagnostic prevalence of ICD-10 diagnoses F12.X (cannabis-related disorders) in insurees aged 12 to 17 years for calendar years 2013–22, stratified by age group and sex. From 2013 to 2022, the diagnostic prevalence of cannabis-related disorders among adolescents utilizing outpatient healthcare services increased from 0.08% to 0.10% (+22.4%), with higher diagnostic prevalence in older adolescents. Overall, 78.3% of adolescents with a diagnosed cannabis-related disorder had at least one co-occurring child and adolescent psychiatric disorder diagnosis in 2022, with the most common being depressive disorders, conduct disorders, adjustment disorders, attention-deficit/hyperactivity disorder, and anxiety disorders. This study underscores the urgent need for integrated treatment approaches addressing both cannabis-related disorders and depressive disorder in adolescents.
