# Supplementary material for: Trends in the diagnostic prevalence of cannabis-related disorders and co-occurring psychiatric disorders in adolescents: analysis of German health insurance data from 2013 to 2022
Source: Eur J Public Health. 2026 Jan 9;36(2):ckaf228. doi: 10.1093/eurpub/ckaf228 (PMC13064856; doi:10.1093/eurpub/ckaf228)
Supplement: ckaf228_Supplementary_Data [file ckaf228_supplementary_data.docx]

**Trends in the diagnostic prevalence of** **cannabis-related disorders and co-occurring psychiatric disorders in adolescents:**

**analysis of German health insurance data from 2013–2022**

**Supplementary Materials**

Alexander Zarour^1^, Christian Bachmann^2^, Lisa Dandolo^1^, Jakob Holstiege^3^, Falk Hoffmann^4^, Constanze Scholman^1^, Yulia Golub^1^

*1. Department of Child and Adolescent Psychiatry, Psychosomatic and Psychotherapy, School of Medicine and Health Sciences, Carl von Ossietzky Universität Oldenburg, Oldenburg, Germany*

*2. Department of Child and Adolescent Psychiatry, University Hospital Ulm, Germany*

*3. Central Research Institute of Ambulatory Health Care in Germany, Department of Epidemiology and Healthcare Atlas, Berlin, Germany*

*4. Division of Outpatient Care and Pharmacoepidemiology, Department of Health Services Research, Carl von Ossietzky Universität Oldenburg, Oldenburg, Germany*

Corresponding author: Prof. Dr. Yulia Golub, Klinikum Oldenburg AöR, Department of Child and Adolescent Psychiatry, Psychosomatics and Psychotherapy, University Hospital, Brandenburgerstr. 44, 26133 Oldenburg, Germany, yulia.golub@uol.de , Tel: 0441 403-10061


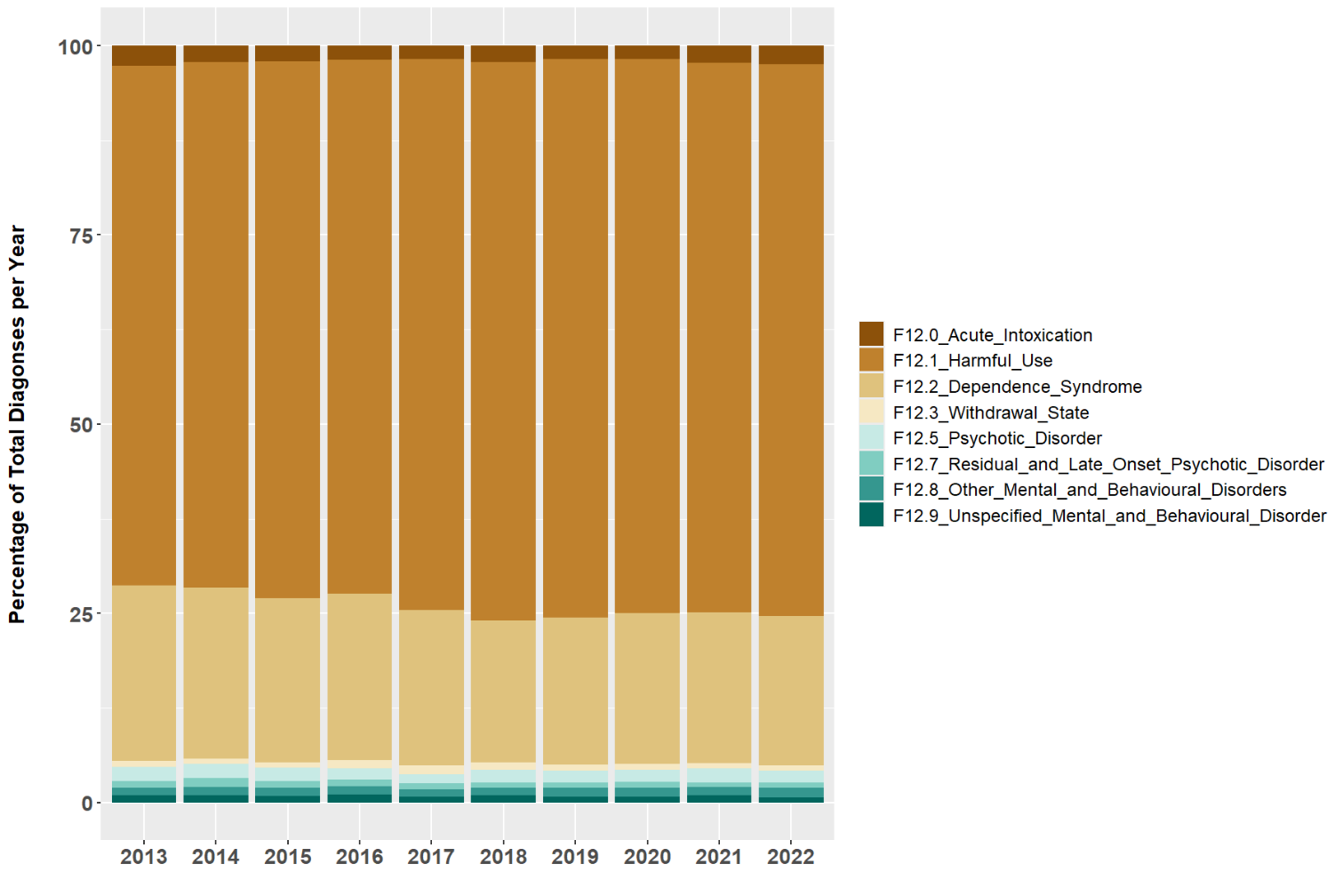


**Figure S1:** For each year from 2013 – 2022 the percentage of F12 subdivision diagnoses is displayed for the subdivisions F12.0 (Acute intoxication), F12.1 (Harmful use), F12.2 (Dependence syndrome), F12.3 (Withdrawal state), F12.5 (Psychotic disorder), F12.7 (Residual and late-onset psychotic disorder), F12.8 (Other disorders), and F12.9 (Unspecified disorder). Note that the number of diagnoses of F12.4 (Withdrawal state with delirium) and F12.6 (Amnesic syndrome) were too small to report for data protection reasons. The underlying data for this figure includes all F12 diagnoses per year and is not directly related to the number of patients with each diagnosis, as e.g. a patient might be diagnosed with the same ICD-10 code in more than one quartal. The far most common diagnosed subdivision is F12.1 (Harmful use) with percentages ranging from 68.6 % in 2023 to 73.8 % in 2018. The second most common diagnosis in each of the years was F12.2 (Dependence syndrome), ranging from 18.7% in 2018 to 23.2 % in 2013.


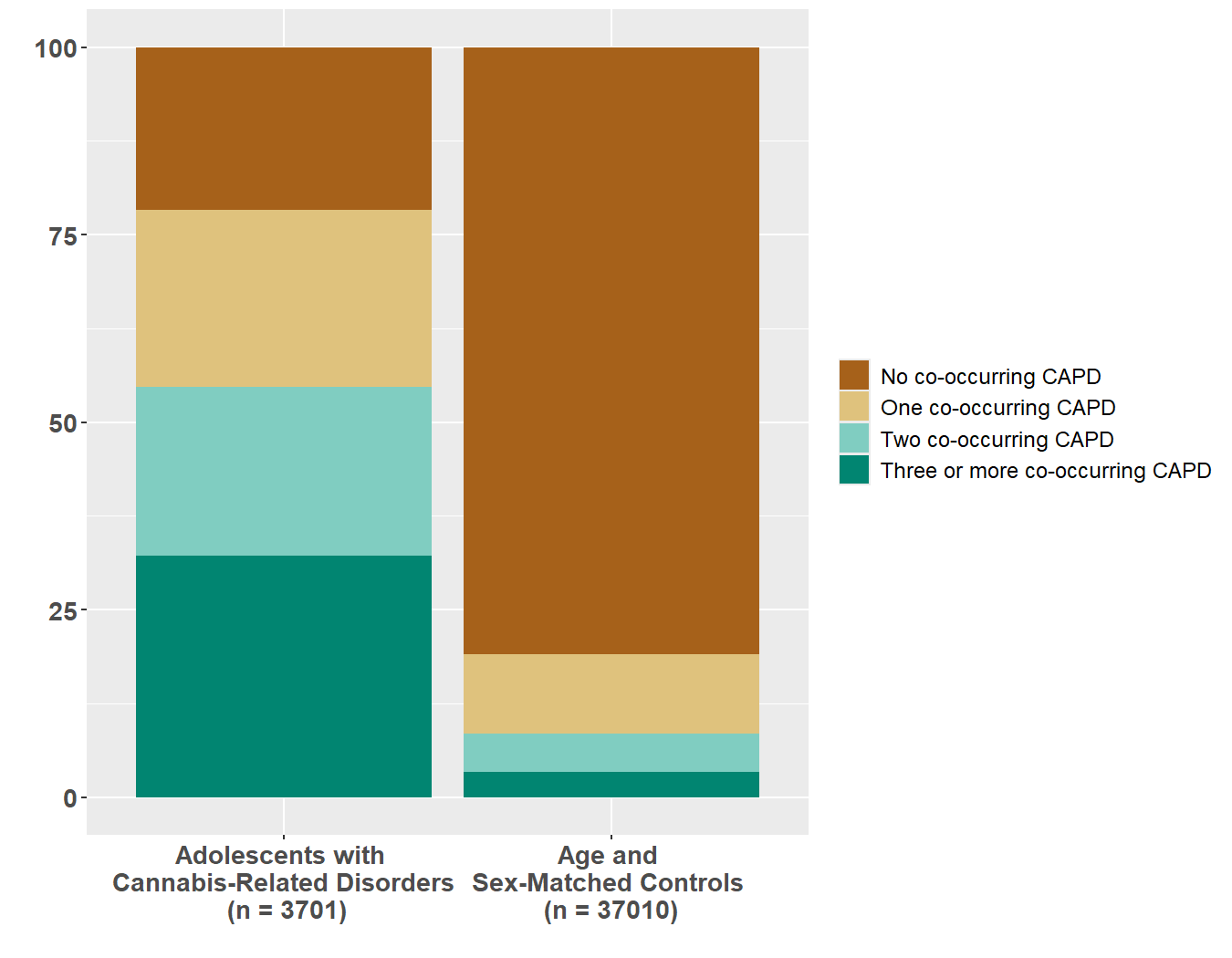


**Figure S2:** The percentage of adolescents with either no co-occurring CAPD, one co-occurring CAPD, two co-occurring CAPD or three or more co-occurring CAPD in the group of adolescents with cannabis-related disorders (n = 3,701) and the 1:10 age and sex-matched control group (n = 37,010) in the year 2022. Within the group of adolescents with cannabis-related disorders most patients had three or more co-occurring CAPD (32.2%), with the rest of the adolescents in this group evenly distributed amongst the counts for no (21.7%), one (23.6%) or two (22.5%) co-occurring CAPD. Within the age and sex-matched control group, by far the most adolescents had no CAPD (80.9%), followed by 10.6% with one CAPD, 5.2% with two co-occurring CAPD and only 3.3% with three or more co-occurring CAPD.


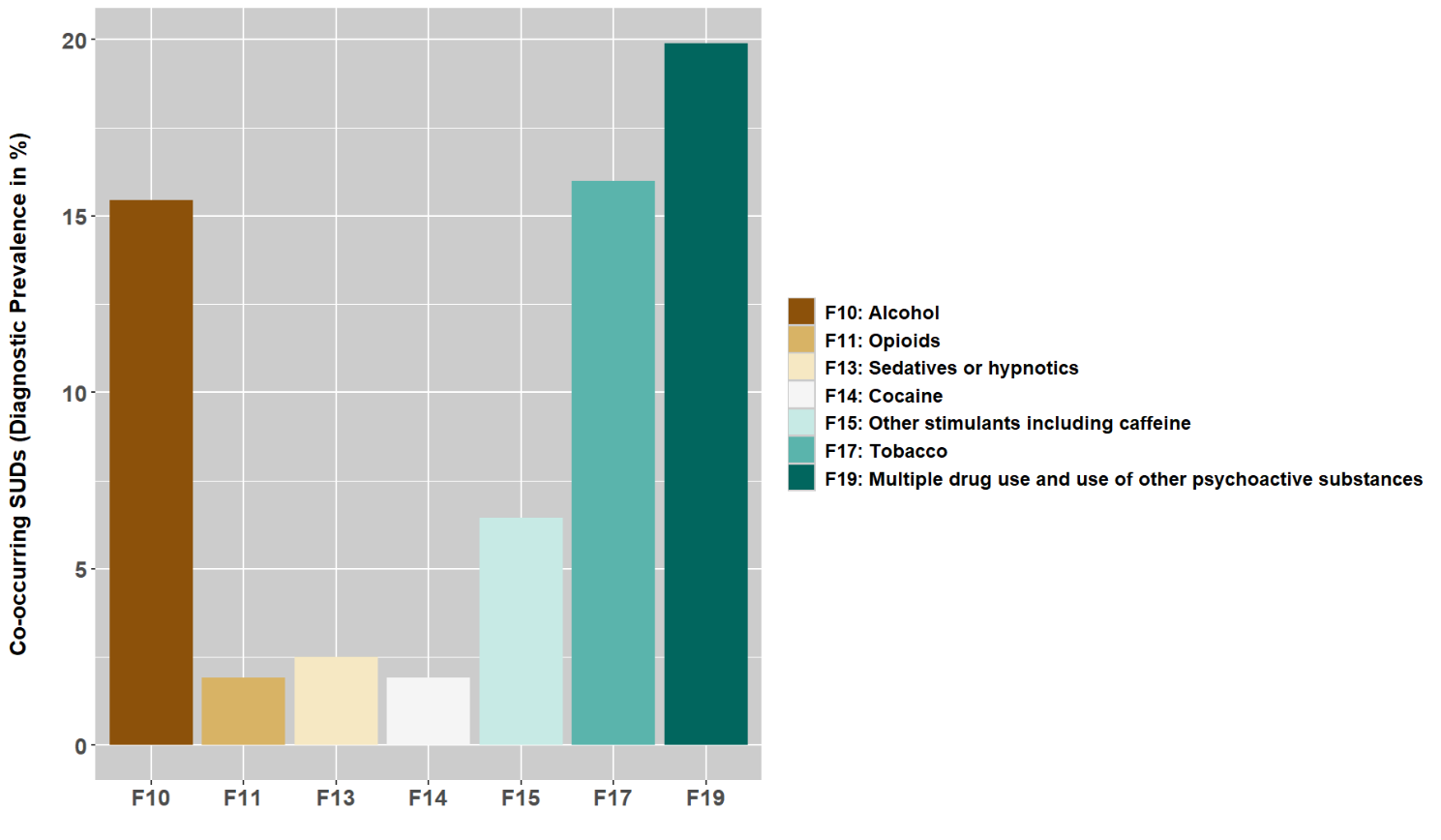


**Figure S3:** Diagnostic prevalence of other co-occurring substance related disorders within the group of adolescents with cannabis-related disorders in the year 2022 (n = 3701). Data is shown for the ICD-10 codes for alcohol (F10), opioids (F11), sedatives or hypnotics (F13), cocaine (F14), other stimulants including caffeine (F15), tobacco (F17), and multiple drug use and use of other psychoactive substances (F19). Note that the number of people with co-occurring F16 (hallucinogens) and F18 (volatile solvents) diagnoses were too small to report for data protection reasons.
